# Tiefes periokuläres Trauma mit Tränenwegsbeteiligung durch Kaiserschnittentbindung

**DOI:** 10.1007/s00106-021-01039-8

**Published:** 2021-04-06

**Authors:** Jens Heichel, Hans-Gert Struck, Friedrich Paulsen, Mohammad Javed Ali, Arne Viestenz

**Affiliations:** 1 Universitätsklinik und Poliklinik für Augenheilkunde, Martin-Luther-Universität Halle-Wittenberg, Ernst-Grube-Str. 40, 06120 Halle, Deutschland; 2 Institut für Anatomie, Lehrstuhl II, Friedrich-Alexander-Universität Erlangen-Nürnberg, Universitätsstr. 19, 91054 Erlangen, Deutschland; 3 Govindram Seksaria Institute of Dacryology, L. V. Prasad Eye Institute, 500034 Hyderabad, Indien

**Keywords:** Orbita, Lider, Kinderaugenheilkunde, Tränenwegsintubation, Calamari ring sign, Orbit, Eyelids, Pediatric ophthalmology, Lacrimal intubation, Calamari ring sign

## Abstract

Periokuläre Verletzungen im Rahmen einer Kaiserschnittentbindung sind extrem selten. Anhand einer Kasuistik eines 5 h alten Neugeborenen soll das Trauma-Management in Hinblick auf Diagnostik, Therapie und Nachsorge einer tiefen periokulären Weichteilwunde dargestellt werden. Das Verletzungsmuster umfasste durchgreifende Wunden des medialen und lateralen Lidwinkels mit Eröffnung der Bindehautumschlagsfalte, der Tenonkapsel und Durchtrennung des oberen Tränenröhrchens. Der schichtweise Wundverschluss beinhaltete eine autostabile monokanalikulonasale Intubation der ableitenden Tränenwege.

**Video online:**

Die Online-Version dieses Beitrags (10.1007/s00106-021-01039-8) enthält zwei Videos zum Einführen der Ritleng-Sonde und dem Ausleiten des Führungsfadens. Beitrag und Zusatzmaterial stehen Ihnen auf www.springermedizin.de zur Verfügung. Bitte geben Sie dort den Beitragstitel in die Suche ein, das Zusatzmaterial finden Sie beim Beitrag unter „Ergänzende Inhalte“.

## Abstract (en)

Periocular injuries during a caesarean section are extremely rare. The case report of a five hour old newborn addresses the trauma management concerning diagnostics, therapy, and post-operative care of a deep periocular soft tissue injury. The pattern of injury consisted of full thickness wounds of the medial and lateral lid margins, opening of the superior conjunctival fornix, and penetration of Tenon’s capsule. The reconstruction was performed layer by layer while an autostable monocanaliculonasal lacrimal intubation was inserted.

Periokuläre Weichteilwunden können eine Beteiligung der ableitenden Tränenwege (TNW) aufweisen. Die Rekonstruktion dieser filigranen Strukturen erfordert eine genaue Diagnostik und einen schichtweisen Wundverschluss, bei welchem eine temporäre Schienung der TNW unabdingbar ist [1, 2].

## Anamnese

**Figure 1:**
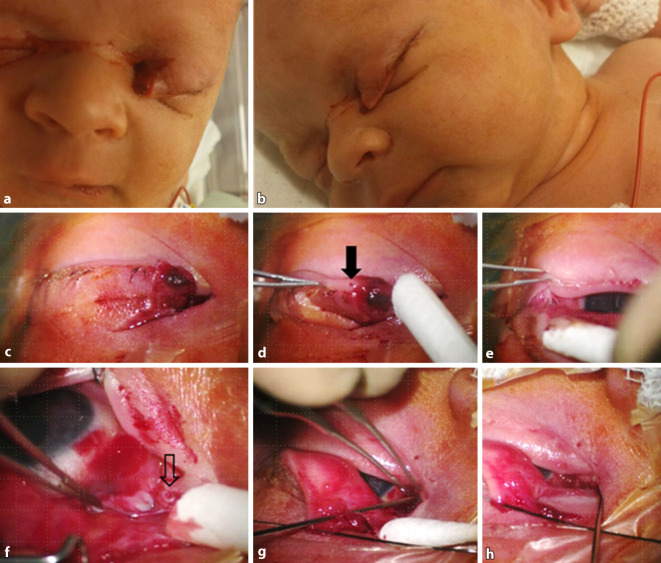

Bei einer 17-jährigen Nullipara entwickelte sich ein Amnioninfektionssyndrom mit auffälliger Kardiotokographie. Es wurde eine notfallmäßige Kaiserschnittentbindung (KSE) durchgeführt. Hier kam es beim Fetus zu einer tiefen Rissverletzung des linken Auges. Aufgrund einer sehr dünnen Uteruswand war es im Rahmen der Uterotomie zu einer starken Blutung gekommen, welche durch eine Kocher-Klemme gestoppt werden sollte. Durch ein Abrutschen während des Platzierens der Klemme wurde das Kind verletzt (Abb. 1). Das 5 h alte Neugeborene wurde sofort in unsere Klinik verlegt.

**Figure 2:**
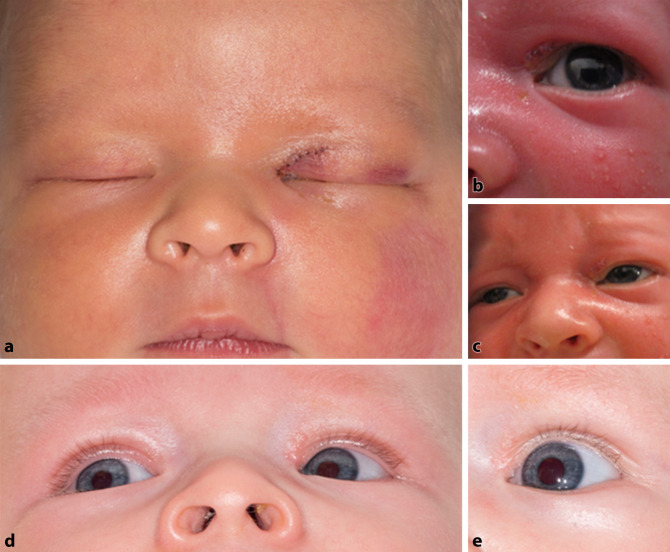

## Befund

Bereits die makroskopische Inspektion ließ eine komplette Durchtrennung des medialen Oberlids erkennen (Abb. 1a, b). Auch eine tiefe Bindehautwunde entlang der nasalen oberen Umschlagsfalte stellte sich dar. Das Kind zeigte eine seitengleich intakte Pupillenreaktion mit Abwehr auf hellen Lichtreiz. Der Augeninnendruck war palpatorisch seitengleich normoton. Handspaltenmikroskopisch bestand kein Anhalt für eine intraokuläre Verletzungsfolge am vorderen Augenabschnitt. Bei der notwendigen Rekonstruktion in Allgemeinanästhesie wurde die Untersuchung des hinteren Augenabschnitts in medikamentös erweiterter Pupille im Rahmen einer präoperativen Narkoseuntersuchung erbracht. Ein Anhalt für ein bulbuseröffnendes Trauma bestand nicht.

Im Weiteren erfolgte intraoperativ eine genaue mikroskopische Untersuchung zur Evaluierung der periokulären Verletzungsfolgen. Neben der Oberlidwunde (Abb. 1c) zeigte sich der Canaliculus lacrimalis superior knapp nasal des Punctum lacrimale superius durchtrennt (Abb. 1d). Der proximale Stumpf des Tränenröhrchens präsentierte sich durch ein „Calamari ring sign“ (Abb. 1f). Die Conjunctiva bulbi und Tenon-Kapsel waren ebenso eröffnet. Die vertikale Ausdehnung des Defekts betrug 12 mm und zog eine Abtrennung des Oberlids vom medialen Lidhalteapparat nach sich. Die obere Umschlagsfalte der Bindehaut war hier über 10 mm eröffnet. Der laterale Lidwinkel zeigte einen Einriss von 6 mm Länge (Abb. 1e).

## Diagnose

Akzidentelle periokuläre Weichteilwunde mit Tränenwegsbeteiligung durch Abriss des medialen Oberlids und Einriss der lateralen Lidkommissur im Rahmen einer KSE.

## Therapie und Verlauf

Unter Einsatz des Operationsmikroskops wurden die TNW durch Vorlegen des Führungsfadens einer autostabilen monokanalikulonasalen Intubation gesichert (Abb. 1g, h). Es folgte ein schichtweiser Wundverschluss mit Adaptation des Tarsus am medialen Lidbändchen, Rekonstruktion des oberen Fornix conjunctivae, Kanalikulus‑, Muskel- und Hautnähte. Für die Naht kam polyphiles resorbierbares Material der Stärke 8‑0 zum Einsatz. Das Kind wurde für 2 Wochen mit antibiotisch-antiphlogistischen Augentropfen für den Bindehautsack und Augensalbe für die Hautbereiche versorgt (Abb. 2).

Das Silikonstützmaterial der TNW wurde nach 8 Wochen entfernt. In der Nachbeobachtung von 6 Monaten fand sich kein Anhalt für eine Störung im Bereich der TNW. Die Lidspalte stellte sich symmetrisch und vollständig beweglich dar und zeigte keinerlei Diskontinuitäten zur Bulbusoberfläche.

## Diskussion

### Risikofaktoren

Kindsverletzungen im Rahmen einer KSE kommen in 0,1–3 % der Fälle vor [3–5]. Direkt periokuläre Manifestationen gelten jedoch als Rarität, und es existieren nur einzelne Fallberichte hierzu.

Verschiedene Faktoren nehmen Einfluss (Indikation zur Entbindung, Erfahrung des Operateurs, mütterliche und kindliche Anatomie, Kindslage, Gestationsalter, etc.) [4, 6]. Eine notfallmäßige Indikationsstellung zur KSE und die Ausdünnung der Uteruswand stellen die wichtigsten Einflussgrößen dar. Je nach Ausgangssituation kann die Wandung der Gebärmutter lediglich 2–3 mm betragen [4]. Zumeist handelt es sich um kosmetisch störende Verletzungen ohne funktionelle Folgen, wobei auch Verletzungen mit Todesfolge beschrieben wurden [7].

### Besonderheiten von Lid- und Tränenwegstraumata

Periokuläre Verletzungen mit Affektion des Lidhalteapparats können zu Lidfehlstellungen führen. Allein hierdurch, aber auch im Fall einer zusätzlichen Verletzung der TNW besteht das Risiko für eine permanente Epiphora [2]. Dies beeinflusst die visuelle Entwicklung eines Kindes. Gerade die Verbindung der Pars lacrimalis (Horner) des M. orbicularis oculi ist für ein einwandfreies Funktionieren der Tränenpumpe entscheidend. Lidhalteapparat, Lidmotorik und TNW stellen eine funktionelle Einheit dar, bei welcher die perikanalikuläre Muskulatur besonderer Berücksichtigung bedarf [1, 2].

Durch die filigrane Anatomie ist der mediale Lidwinkel besonders gegenüber tangentialer Krafteinwirkung exponiert. In über 50 % der Verletzungsfälle ist der untere Kanalikulus betroffen, bei etwa 33 % der obere bzw. 12 % beide Kanalikuli. Eine Mitbeteiligung des Augapfels besteht in 25 %. Dies ist dann zu vermuten (über 83 %), wenn es zu einer Verletzung des oberen Tränenröhrchens gekommen ist. Die tiefer gelegenen Anteile der TNW sind in 20 % (Tränensack) bzw. 10 % (Tränennasengang) affektiert und zumeist Folge schwerer Mittelgesichtstraumata [1, 8].

### Erkennung von Tränenwegsverletzungen

Die intraoperative Erhebung des Verletzungsmusters stellt die Basis für eine adäquate Rekonstruktion der TNW dar. Prinzipiell ist ein Vorgehen in Allgemeinanästhesie vorteilhaft, da eine lokale Anästhesie bei ohnehin schwierigen Wundverhältnissen eine zusätzliche Schwellung des Gewebes mit sich bringen und die Übersicht erschweren kann. Durch die perikanalikuläre Muskulatur zeigen die freiliegenden Kanalikulusstümpfe oft ein auswärts gerolltes Epithel und bilden hierdurch einen wulstigen Schleimhautring („calamari ring sign“) [9]. Auch im hier dargestellten Fall war dies ein wegweisendes klinisches Zeichen.

Zur indirekten Darstellung des verletzten Tränenröhrchens eignen sich folgende Maßnahmen (jeweils Eingabe über eines der Tränenröhrchen und ggf. digitale Kompression des Tränensacks):

- Spülung mit physiologischer Kochsalzlösung;
- Luftinstillation in die TNW;
- Methylenblau;
- Methylzellulose/Viskoelastikum/Hyaluronsäure;
- Fluoreszein 2 %;
- Kombinationen aus oben genannten Maßnahmen (z. B. mit Fluoreszein versetztes Viskoelastikum).

Auch die intraoperative diagnostische Spülung der TNW ist bei Traumata wichtig, um eine Beteiligung tiefer gelegener Strukturen (Tränensack und/oder Tränennasengang) erkennen zu können und ggf. dann die Rekonstruktion vorzunehmen [10]. Prinzipiell ist lediglich die Sicherung der verletzten Anteile erforderlich, um eine unnötige Alteration der TNW und damit eine zusätzliche iatrogen verursachte Stenose zu vermeiden. Es gilt der Grundsatz: „so viel wie nötig, so wenig wie möglich“.

### Möglichkeiten der Tränenwegsschienung

Die TNW-Rekonstruktion bei Verletzungen geht mit einer temporären Schienung der verletzten Abschnitte einher. Zahlreiche Techniken stehen hier zur Verfügung (Tab. 1; [11]). Neben der Ringintubation der Tränenröhrchen sind beispielsweise autostabile (selbsthaltende) monokanalikuläre Stützmaterialien wie der Mini-Monoka® bzw. der Monoka® (Fa. FCI, Paris, Frankreich) als monokanalikulonasale Variante für die Kanalikuli verfügbar. Im Fall einer Durchtrennung der distalen (lateralen) 2 Drittel des Kanalikulus kann eine autostabile monokanalikuläre Schienung angewendet werden (Mini-Monoka®). Liegt eine Durchtrennung des proximalen (medialen) Drittels des Tränenröhrchens vor, ist eine autostabile monokanalikulonasale Intubation (Monoka®) von Vorteil. Ein speziell gefertigtes druckknopfartiges Endstück ermöglicht, dass die Silikonverweilsonde keiner zusätzlichen Nahtfixation bedarf und selbsthaltend in den Tränenpünktchen gesichert wird [12, 13].

**Table 1:** 

Lokalisation der Stenose	Monokanalikulär/monokanalikulonasal	Bikanalikulär/bikanalikulonasal
Tränenröhrchen	Minimonoka® (Fayet & Bernard) Lacrijet® für Tränenwegsverletzungen	Ringintubation (Murube del Castillo) Ringintubationsset („Modell Erlangen“) bikanalikuläres autostabiles Set®
Tränensack und/oder Tränennasengang	Monoka® (Ritleng) Masterka® Lacrijet®	Bikanalikulonasale Intubation (Jünemann) BIKA-Intubationsset® Intubationsset Ritleng+® Nunchaku®
Osteotomie im Rahmen einer DZR	Minimonoka® (Fayet & Bernard) Monoka® (Ritleng) Mono-Crawford®	Bikanalikulonasale Intubation (Crawford) Nunchaku®
Konjunktiva/Nase im Rahmen einer Konjunktivorhinostomie	Lester-Jones-Tube Tube de Metaireau®	–

*DZR* Dakryozystorhinostomie, ® FCI, Paris, Frankreich

Der Vorteil autostabiler monokanalikulärer/monokanalikulonasaler Stützmaterialien bei Kleinkindern (und Säuglingen) ist hier, dass ein weiteres Wachstum während des Verweilens der TNW-Schienung möglich ist und somit weniger Komplikationen wie Einrisse der Tränenpünktchen oder Schlauchdislokationen zu erwarten sind. Im hier dargestellten Fall wurde eine Entscheidung für die TNW-Rekonstruktion mittels autostabiler monokanalikulonasaler Intubation in Ritleng-Technik (Monoka®) getroffen (Video 1 und 2) [13]. Eine Ringintubation wäre vermutlich infolge des zu erwartenden Wachstums des Kindes mit einer Alteration der Tränenröhrchen durch Dehnung und Schlitzung einhergegangen. Die Verwendung eines Mini-Monoka® wäre aufgrund fehlender Sondenführung des Stützmaterials bei entsprechend engen anatomischen Verhältnissen nur schwer zu platzieren gewesen. Auch die bikanalikulonasale Intubation („U-Intubation“) gewährleistet eine effektive Schienung der verletzten TNW. Wird die Spannung gut dosiert, ermöglicht die Technik eine zusätzliche Stützung des medialen Lidwinkels und führt zu funktionellen Erfolgsraten von ca. 85 %. Die intubatspezifischen Komplikationen liegen bei etwa 17 % (4 % Intubatdislokation, 3 % Epiphora durch mechanische Alteration der Augenoberfläche, 2 % „cheesewiring“ der Tränenpünktchen, 1 % Granulombildung) [14].

### Zeitpunkt der Rekonstruktion

Generell spielt der Allgemeinzustand des Patienten eine maßgebende Rolle. Besteht eine vitale Bedrohung oder liegt ein offenes Bulbustrauma vor, müssen diese Verletzungsfolgen priorisiert werden. Existieren lediglich Weichteilverletzungen der okulären Adnexe ohne erhebliche Blutung oder Visusbedrohung und gibt es keinen Anhalt für eine Wundkontamination, muss die Rekonstruktion nicht dringlich erfolgen. So kann beispielsweise bis zu 48 h gewartet werden, falls keine entsprechend versierten Operateure verfügbar sind. Ideal sind dennoch Zeiträume von 6 bis 8 h, da der posttraumatische Gewebeumbau noch nicht wesentlich ist [1].

Bleibt die Rekonstruktion der TNW aus, kommt es zu einer vollständigen Vernarbung. Eine sekundäre Rekonstruktion gestaltet sich zumeist schwieriger und erfordert häufig mehrere, zum Teil komplexe, chirurgische Interventionen.

## Fazit für die Praxis

- Eine KSE birgt das Risiko schwerer periokulärer Verletzungen.
- Die adäquate Versorgung ermöglicht eine Rekonstruktion unter Berücksichtigung funktioneller und ästhetischer Gesichtspunkte.
- Die autostabile monokanalikulonasale Intubation nach Ritleng eignet sich auch für TNW-Verletzungen bei Neugeborenen.
- Autostabile TNW-Intubationen eignen sich gut für ein größendynamisches System wie das eines heranwachsenden Säuglings.
- Die Versorgung schwerer Lidverletzungen erfordert eine schichtweise Rekonstruktion der Gewebe. Sie erfolgt aus der Tiefe heraus und von medial nach lateral.
- Voraussetzung hierfür ist die exakte Identifikation und Zuordnung der betroffenen anatomischen Strukturen.
- Tränenröhrchenverletzungen können durch das „calamari ring sign“ erkannt werden.

## Supplementary Information





